# Enabling meaningful participation and involvement in oncology and palliative care research: insights from two participatory research projects

**DOI:** 10.1186/s12904-026-02415-3

**Published:** 2026-11-04

**Authors:** Till Neugebauer, Stephanie Knippschild, Veronika Bencheva, Sven Schmiedl, Fabian Erdsiek, Tuğba Aksakal, Yüce Yılmaz-Aslan, Patrick Brzoska

**Affiliations:** 1 Health Services Research, School of Medicine, Faculty of Health, Witten/Herdecke University, Witten, Germany; 2 Institute for Medical Biometry and Epidemiology, School of Medicine, Faculty of Health, Witten/Herdecke University, Witten, Germany; 3 Clinical Pharmacology & Center for Clinical Trials, School of Medicine, Faculty of Health, Witten/Herdecke University, Witten, Germany; 4 Philipp Klee-Institute for Clinical Pharmacology, Helios University Hospital Wuppertal, Wuppertal, Germany

**Keywords:** Patient and public involvement, Adaptive strategies, Co-design, Evaluation, Participation

## Abstract

**Background:**

Patient and public involvement (PPI) is increasingly recognized as essential in oncology and palliative care research. Yet, achieving meaningful participation of diverse stakeholders in multidisciplinary research teams remains difficult. This study aimed to explore how participatory processes were shaped, experienced, and adapted across two research projects in Germany, to identify aspects that facilitated or hindered meaningful participation and involvement and how collaboration is perceived.

**Methods:**

A retrospective qualitative process evaluation of participatory processes across two research projects in oncology and hospice and palliative care was conducted. These projects involved patients, relatives, patient representatives, and healthcare professionals as co-researchers alongside academic researchers. Data included reflective group discussions, workshop and meeting documentation, co-researchers’ feedback, and researcher reflections. Data were analyzed inductively using thematic analysis and thematic triangulation across data sources and project contexts.

**Results:**

Three overarching themes were identified: (1) facilitating meaningful participation and involvement, (2) challenges and adaptive strategies in the participatory process, and (3) perceived impacts of participation. Early involvement, transparent communication, visible use of contributions, and accessible research tasks supported trust, ownership, and perceived relevance. Challenges included changing participation capacities, scientific and epistemic complexity, digital and accessibility barriers, communication difficulties, and power imbalances. Flexible scheduling, facilitated working sessions, accessible tools, stronger emphasis on verbal feedback, and active moderation supported continued participation. Co-researchers described increased confidence, research understanding, and a sense of agency, while researchers perceived improvements in the relevance and usability of research processes and outputs.

**Conclusions:**

Meaningful participation and involvement depended on combining clear structures with sufficient flexibility to respond to changing capacities, knowledge levels, and relational dynamics. Participatory processes in oncology and palliative care may therefore benefit from adaptable approaches that support sustained influence without requiring uniform or continuous levels of participation.

**Supplementary Information:**

The online version contains supplementary material available at https://doi.org/10.1186/s12904-026-02415-3.

## Introduction

Participatory approaches, such as patient and public involvement (PPI), have gained relevance in health services research, especially within oncology care pathways that span curative, palliative, and hospice settings [1, 2]. PPI seeks to systematically integrate the perspectives, experiences, and needs of individuals with lived experience into all stages of the research process [3]. In contrast to traditional research paradigms, which often position patients and other stakeholders as passive subjects, participatory approaches aim to involve them as active partners or co-researchers [4]. In the United Kingdom, PPI has become an integral component of the National Health Service, shaping research agendas and outcomes [5]. In Germany, however, the implementation of such approaches is still emerging [6]. Nevertheless, participatory research has gained increasing attention in recent years, particularly within the field of participatory health research, which aligns with the principles of PPI. Participation is commonly understood as a process of progressively sharing decision-making power between researchers and those affected by the research topic, with the aim of enabling meaningful participation and involvement throughout the research process [7]. In this context, “meaningful” refers to the perceived relevance of participation and goes beyond symbolic consultation. It requires that patients and the public contribute experiential knowledge, influence research processes and decisions, develop a sense of agency, and become co-owners of the research [7]. This article draws on this understanding to describe the involvement of patients and other stakeholders as co-researchers.

PPI is particularly relevant along the oncology care pathway, where care is inherently complex, multidisciplinary, and closely tied to patients’ individual life contexts [1]. Conventional top-down research designs frequently fail to capture the lived experiences and priorities of affected individuals, especially those​ in vulnerable situations or underserved populations [8]. These include, for example, individuals with advanced illness, limited health literacy, low socioeconomic status or structurally disadvantaged groups, whose perspectives are often underrepresented in research processes [9, 10]. By actively involving patients and the public as co-researchers in the production of knowledge, participatory research has the potential to enhance the relevance, acceptability, and implementation of research outcomes [11]. Depending on the aim of the research, co-researchers do not necessarily have to be individuals contributing primarily through personal lived experience, such as patients or relatives. Healthcare professionals, including physicians and nurses, may also act as co-researchers by contributing experiential and contextual knowledge from clinical practice [12]. This broader understanding of PPI reflects the multidisciplinary nature of care along the oncology pathway and allows for the integration of complementary perspectives within research.

Despite its conceptual advantages, the implementation of participatory approaches remains challenging [13]. Barriers include differences in knowledge and expectations between researchers and co-researchers, role ambiguities, communication challenges, and structural constraints within research environments [14]. Without careful consideration of these challenges, participatory processes may remain tokenistic, meaning that patients and other stakeholders are included primarily to fulfill formal expectations rather than to actively shape research processes and decisions [4]. A “one-size-fits-all” [15] strategy is therefore unlikely to be appropriate to address this heterogeneity of challenges and underscores the importance of targeted and tailored approaches to ensure flexibility within participatory processes [16].

Despite increasing recognition of PPI, evidence on how participatory approaches can be implemented in multidisciplinary co-researcher groups remains limited, particularly in palliative care and oncology research. Existing studies predominantly focus on the involvement of patients and family members, while less attention has been paid to participatory settings that also include healthcare professionals [17, 18]. Bringing together stakeholders with different forms of experiential, professional, and scientific knowledge may create distinct participation dynamics and power relations that are not yet well understood.

To address this gap, the present study explores the following research question: How were participatory processes shaped, experienced, and adapted in two research projects in Germany, and which aspects facilitated or hindered meaningful participation and involvement?

## Methods

### Design

This study was a retrospective qualitative process evaluation of participatory processes embedded within two independently conducted research projects from a hospice and palliative care setting, as well as an oncological research field in Germany. The evaluation explored how participation was experienced, shaped, and adapted in practice, including the collaboration between researchers and co-researchers, barriers and enabling conditions encountered, and perceived impacts of participation.

The participatory approach of both projects was conceptually informed by principles of participatory health research, particularly shared influence, co-creation of knowledge, and active engagement in the research process [7]. These principles guided the understanding and design of participation but were not used as predefined evaluation criteria or as an analytical framework. Instead, the evaluation followed an exploratory, inductive approach. At the beginning of both projects, the roles of co-researchers and mutual expectations regarding collaboration were explicitly introduced and discussed.

Throughout this manuscript, "co-researcher" refer to patients, relatives, patient representatives, and healthcare professionals actively involved in research activities within the projects described, whereas members of the academic project teams are referred to as "researchers". The evaluation encompassed the participatory activities of both projects, with co-researchers and researchers contributing through direct reflections, feedback, and documented collaborative activities. Ethical approval for the evaluation of both projects was obtained from the ethics committee of Witten/Herdecke University (S-222/2025). The reporting of PPI activities was informed by the Guidance for Reporting Involvement of Patients and the Public (GRIPP2) checklist [19]. An offline version of the large language model GPT-OSS-20B (OpenAI), hosted on a local server and accessed via an Ollama interface, was used to edit the manuscript’s language as part of the review process. The content was reviewed and revised. The authors take responsibility for the quality of the content. No initial drafts, analyses, or interpretations were generated by artificial intelligence.

### Project contexts and co-researchers

SETT-AID (“Sex-specific differences in Efficacy and Tolerability of novel T-cell-targeted Immunotherapeutic antineoplastic Drugs”), funded by the Federal Ministry of Research, Technology, and Space in Germany (Grant number: 01GN2526), examines sex differences in the efficacy, safety, and patient-reported outcomes of novel oncological therapies by conducting systematic reviews and individual participant data meta-analyses [20, 21]. The project began in 2025 and concluded its main participatory activities by October 2026. In total, 20 patients undergoing cancer treatment, patient representatives, and individuals with chronic conditions were involved as co-researchers through two workshops in which they discussed and prioritized patient-relevant outcomes and contributed to the interpretation and contextualization of findings. Each workshop lasted about two and a half hours. Two patients were also involved in conducting the systematic reviews, including literature screening, selecting studies and extracting data. This assignment included additional meetings with the researchers and a time commitment of two to four hours per week, scheduled individually. The workshops and meetings featured content-related insights as well as active work sessions and group discussions.

ParDiMi (“Participatory development of a manual for the implementation of diversity-sensitive hospice and palliative care”), funded by the German Cancer Aid (Grant number: DKH70115264) over a period of 33 months (October 2023 to June 2026), aimed to develop a practice-oriented manual to support the implementation of diversity-sensitive hospice and palliative care in Germany [22]. After initially inviting 11 patients, relatives, and staff from palliative or hospice care facilities​, nine persons contributed to the identification of relevant topics as co-researchers, while five of the co-researchers contributed to the structure and content of the manual, and the revision of draft materials through a series of nine workshops and two additional meetings. Each workshop lasted about two to three hours. These sessions included, among other things, content-related input on the topic, active work sessions, discussion groups, and presentations by outside experts. Six co-researchers withdrew from the study over time due to health and time constraints. An advisory board of five experts from research and practice, including public health researchers, representatives of palliative care societies, and palliative care specialists, provided ongoing feedback throughout the project. The full study design, including details of the participatory approach, has been published previously [22].

In both projects, co-researchers were purposively recruited through patient organizations, self-help networks, clinical contacts, and direct outreach based on relevant lived or professional experience. Prior to active participation, they received training in the basics of scientific work and the relevant methods specific to the research projects during an introductory session. Co-researchers received financial compensation. In SETT-AID, workshop participants received vouchers and the two longitudinally involved co-researchers were compensated based on the time invested; in ParDiMi, co-researchers received fixed compensation per workshop. No separate sample was recruited for the process evaluation.

The inclusion of these two projects with their distinct methodological objectives for the present study was intentional. While SETT-AID focused on evidence synthesis and involved co-researchers in tasks such as outcome prioritization, literature screening, and interpretation of findings, ParDiMi centered on the co-design of a practice-oriented implementation manual. Comparing participatory processes across these contrasting contexts allowed us to explore whether challenges, facilitating factors, and adaptive strategies were specific to particular research tasks or reflected more general characteristics of PPI in oncology and palliative care research.

### Data sources and collection

Multiple qualitative data sources were used to evaluate participatory processes from the perspectives of both co-researchers and researchers. Across both projects, data included reflective group discussions, process documentation, and observations.

In SETT-AID, data were generated through two Zoom-based reflective group discussions with co-researchers, conducted after the introductory workshop and during the systematic review process. The first discussion included ten co-researchers, while the second included the two co-researchers involved longitudinally in the review process. Both discussions were moderated by the first author, who had not previously worked with the co-researchers within the project. The sessions lasted approximately 20 min. A flexible discussion guide comprising three open-ended questions was used to stimulate conversation while allowing co-researchers to introduce issues they considered relevant. To support the exploratory discussion, the open-ended questions included in the Patient and Public Engagement Evaluation Tool (PPEET) [23] were used as an orientation when developing the discussion guide. The PPEET itself was not administered as a questionnaire or used as a standardized evaluation instrument. The discussions were not audio-recorded. Instead, detailed written notes were taken during and immediately after each session, with selected statements documented verbatim. Researchers subsequently completed structured reflection notes on the participatory process. No formal assessment of data saturation was undertaken because the discussions were designed as project-specific reflective evaluations rather than as stand-alone qualitative interviews.

In the ParDiMi project, data were derived from nine workshops and two additional project meetings conducted between 2023 and 2026. The workshops lasted approximately two hours and were held online. Each meeting was documented by two researchers using an open protocol template. Protocols were prepared during or immediately after the sessions and distinguished between direct contributions from co-researchers, researcher observations of participation dynamics, and subsequent analytical reflections. Where possible, key statements were recorded verbatim; otherwise, contributions were summarized while preserving their intended meaning. Verbal feedback was obtained spontaneously during discussion. Protocols were reviewed by the research team after completion and shared with co-researchers to improve accuracy and contextual interpretation. All co-researchers and researchers contributing data to the process evaluation were informed about the evaluation procedures and the use of their data and provided written consent.

### Analytical approach

A thematic approach was applied to analyze the data, integrating findings from multiple data sources through thematic triangulation [24]. The aim of triangulation was not only to combine different types of data, but also to jointly consider the perspectives of co-researchers and researchers as part of a shared participatory process. This made it possible to examine how opportunities and challenges emerged through the interaction between both groups within the respective project contexts.

All available materials were first reviewed and organized. This was followed by open coding, during which initial themes were inductively generated in light of the research question. In a subsequent step, themes were refined, compared across data sources, and consolidated into an overarching category system. The final category system comprised multiple main- and subthemes that were condensed to three overarching themes to describe them in the results: (1) facilitating meaningful participation and involvement, (2) challenges and adaptive strategies in the participatory process, and (3) perceived impacts of participation (see Additional file 1). Particular attention was paid to preserving contextual nuances, given the heterogeneous nature of the materials. While group discussions primarily captured individual experiences and reflections, observational and process-related data were especially useful for understanding interactional dynamics, structural conditions, and researchers’ adaptive responses during the participatory process.

## Results

### Facilitating meaningful participation and involvement

Across both projects, PPI was implemented early in the research process and extended beyond consultation on final outputs. Co-researchers contributed to ongoing research activities and were given opportunities to shape project developments throughout multiple phases of the studies. Although the form and intensity of involvement differed between projects, both projects aimed to move beyond symbolic participation by enabling co-researchers to influence research priorities, materials, and decisions.

Co-researchers across both projects expressed strong intrinsic motivation to participate. Their motivation was closely linked to the perceived societal relevance of the research, the opportunity to improve future care, and the possibility of contributing experiential knowledge that they felt was often underrepresented in conventional research. Early involvement and visible uptake of their feedback strengthened trust in the research process and reduced perceptions of tokenistic participation.

“I find it very valuable to involve patients from the beginning, because medicine can be incredibly hard to make sense of. Sometimes decisions are made that are simply not transparent or understandable. To build trust, it is important that patients are genuinely listened to.” (Patient co-researcher, SETT-AID).

Experiences of collaboration were largely positive across both projects. Co-researchers described interactions with researchers as respectful, transparent, and supportive. They emphasized the importance of being taken seriously, seeing their contributions reflected in project activities, and being able to ask questions freely. Accessible explanations, opportunities for repetition, and peer support further contributed to confidence, motivation, and perceived self-efficacy.

At the same time, participation did not evolve linearly. In the ParDiMi project, initial uncertainty regarding scientific tasks gradually gave way to stronger identification with the project, whereas in SETT-AID some co-researchers reported a growing sense of ownership that led them to engage beyond their formally assigned tasks. However, researchers also observed limitations in sustained engagement between workshops, particularly regarding the review and revision of written documents. Feedback from co-researchers suggested that competing responsibilities, limited time resources, and health-related burdens often restricted their capacity for additional tasks outside scheduled meetings.

“Unfortunately, six of the co-researchers who initially participated had to withdraw from the study because their health no longer allowed them to continue or because they decided not to spend the final months of their lives participating in a research project.” (Researcher, ParDiMi).

Engagement also varied depending on the type of research task. In the ParDiMi project, co-researchers sometimes found it difficult to envision the final product. Because the final form of the manual was not yet clearly visible, co-researchers sometimes found it difficult to relate individual writing tasks to the overall project goal. This may have reduced engagement in document-based activities between workshops. In contrast, SETT-AID provided more clearly defined research tasks, such as literature screening and data extraction. These findings indicate that meaningful participation depended not only on individual capacities but also on the extent to which research tasks were concrete, transparent, and adaptable to co-researchers’ circumstances.

### Challenges and adaptive strategies in the participatory process

Several interconnected challenges emerged across both projects. A central challenge concerned the scientific complexity of the tasks. Co-researchers reported difficulties understanding methodological procedures, applying inclusion and exclusion criteria, and interpreting scientific literature. In the SETT-AID project in particular, title and abstract screening and the appraisal of English-language articles were described as demanding. These difficulties were not limited to language itself but also related to the complexity of the research topic (novel immunotherapeutic drugs), scientific terminology, study designs, and methodological concepts. Researchers, in turn, emphasized the effort required to translate scientific processes into accessible formats while maintaining methodological rigor.

Technical barriers represented a second major challenge. Differences in digital skills, familiarity with software applications (e.g., using Microsoft Office applications [Excel] or the evidence synthesis tool CADIMA), and preferred devices (smartphone vs. laptop) affected participation in tasks requiring shared document editing or engagement with project tools.

“I don’t do this kind of work on my phone. I need to use my laptop, which means I can only work on my tasks when I’m at home. The larger screen simply makes it much easier for me.” (Patient co-researcher, SETT-AID).

In some cases, access to online documents was hampered by privacy settings or a lack of confidence in using digital tools. Researchers therefore had to make frequent adjustments during meetings and​ adapt workflows so that co-researchers could contribute meaningfully despite these barriers.

A third challenge concerned communication and participation dynamics. Some co-researchers criticized irregular communication, including delays in project updates, late announcements of meetings, and limited feedback on how their contributions had been incorporated. In addition, power imbalances between different groups of co-researchers became visible in collaborative meetings, particularly when healthcare professionals, and especially physicians, dominated discussions. Researchers responded by actively moderating meetings in order to create more equitable communicative structures and to ensure that patient and relative perspectives were not marginalized.

In response to these challenges, both projects introduced adaptive strategies to sustain participation. These included flexible scheduling of meetings and training sessions, facilitated writing sessions in which researchers guided co-researchers through tasks in real time, and the use of more accessible digital tools. In ParDiMi, document-sharing procedures were simplified to avoid account-based barriers. In both projects, expectations regarding written contributions were adjusted where necessary, and participation was increasingly focused on verbal feedback, joint discussion, and decision-making when this was more feasible for co-researchers.

“I think this concept works well because patients are listened to and involved. It is important to receive feedback and know what has happened with your contributions rather than feeling that they disappear into a black hole. Being informed about how feedback is used gives you the feeling that your voice is genuinely being heard.” (Health professional co-researcher, ParDiMi).

Automated translation tools and peer support also served as pragmatic strategies to address linguistic and technical challenges. Although these adaptations often increased the workload for researchers, they were considered essential for maintaining meaningful involvement under real-world conditions. Table 1 summarizes the main challenges, strategies, and implications for participation and co-design identified across both projects.

**Table 1:** Challenges, adaptive strategies, and contributions to participation and involvement in oncology and palliative care research; experiences from the projects SETT-AID and ParDiMi

Challenge/barrier	Adaptive strategy used	Contribution to participation and involvement
Changing participation capacities	• Flexible scheduling • Adaptation of workload • Reduced expectations regarding continuous written work	Enabled participation according to individual capacities and supported continued involvement in vulnerable contexts.
Unfamiliarity with research	• Introductory workshops • Methodological explanations • Repeated clarification • Regular training sessions	Strengthened understanding of research tasks and enabled co-researchers to contribute more confidently.
Scientific complexity	• Preparation of specific task packages • Guided discussions • Facilitated working sessions	Made complex research tasks more accessible and supported meaningful contributions to review and development processes.
Accessibility barriers	• Use of automated translation tools • Peer support • Verbal explanation of scientific content	Improved access to scientific materials while highlighting the need for epistemically accessible research processes.
Digital barriers	• Use of more accessible digital tools • Hands-on software training​ • Real-time support • Adaptation of document-sharing procedures	Reduced exclusion from document-based collaboration and enabled broader participation in revisions.
Sustaining engagement	• Shorter and more manageable tasks • Facilitated online working sessions • Stronger focus on verbal feedback during meetings	Shifted participation toward formats that were more feasible for co-researchers and reduced burden.
Communication challenges	• Regular email updates • Summaries of revisions • Clearer communication of how contributions were used	Strengthened transparency, trust, and continuity of engagement.
Power imbalances between co-researchers	• Active moderation of discussions • Explicit efforts to create equitable communication structures	Supported the inclusion of patient and relative perspectives and prevented domination by professional voices.
Co-design challenges	• Joint prioritization of topics • Collaborative revision of materials • Shared decisions on structure, content, layout, and dissemination	Supported co-design of project outputs and increased perceived relevance, usability, and ownership.

### Perceived impacts of participation

Across both projects, participatory collaboration generated perceived benefits at both the individual and process level. For co-researchers, participation was associated with increased confidence, improved understanding of scientific processes, and a growing sense of competence in contributing to research-related tasks.

“The more involved I became, the more I enjoyed it and the more I wanted to continue being part of it.” (Patient co-researcher, SETT-AID).

These experiences were described as empowering and contributed to co-researchers’ willingness to remain involved in future research. For researchers, co-researcher input improved the relevance and usability of research materials and processes, even where participation required substantial adaptation and additional effort.

**Fig. 1:**
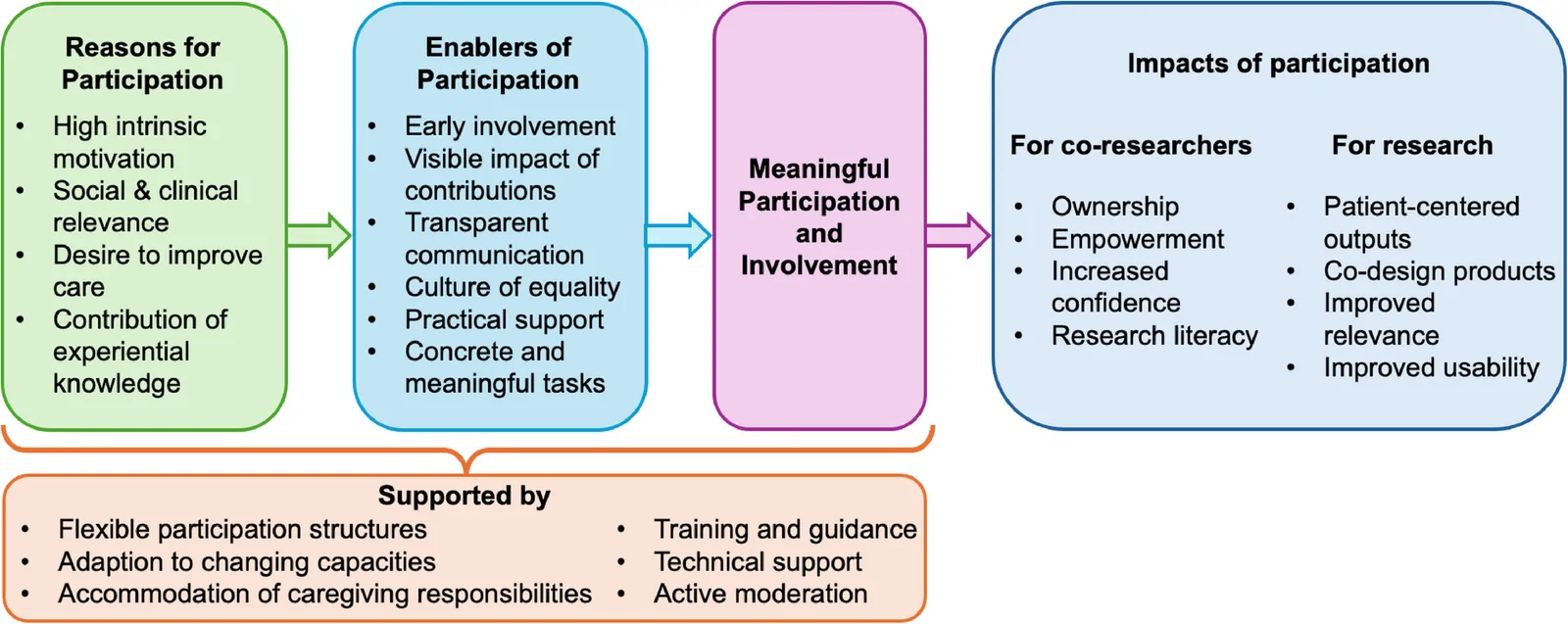
Synthesis of findings on participation and involvement in oncology and palliative care research based on​ experiences from the projects SETT-AID and ParDiMi. Own illustration

Taken together, our findings suggest that meaningful participation and involvement depend on combining clear structures with sufficient flexibility to respond to changing capacities and needs. To illustrate this, Fig. 1 summarizes the key findings across both projects and illustrates how reasons for participation, enabling aspects, and supportive conditions contributed to meaningful participation and involvement, as well as the perceived impacts for co-researchers and research processes in oncology and palliative care research.

## Discussion

This study explored how PPI was shaped, experienced, and adapted across two participatory research projects in hospice and palliative care as well as oncology settings in Germany. The findings show that involving patients, relatives, and other stakeholders as co-researchers was perceived as meaningful by both researchers and co-researchers and contributed to more patient-centered research processes. At the same time, the study highlights that meaningful involvement in contexts characterized by serious illness, fluctuating capacities, and complex care needs requires substantial methodological, structural, and relational adaptations.

A central finding of this study was the high intrinsic motivation of co-researchers to participate in research. Co-researchers described their involvement as an opportunity to contribute lived experiences, improve future care and address perspectives that they perceived insufficiently represented in conventional research. This aligns with previous PPI research showing that public contributors are often motivated by altruistic intentions, personal experiences with healthcare systems or the desire to improve care for future patients [25, 26]. In our projects, early involvement and transparent communication were particularly important, as they strengthened trust, fostered ownership, and reduced perceptions of tokenistic participation [4].

At the same time, our findings suggest that traditional expectations of continuous and highly active participation may be difficult to sustain along the oncology pathway. Patients and relatives frequently described uncertainty regarding their future health situations and questioned whether they could reliably remain involved throughout longer project periods. This reflects a challenge that has received limited attention in existing PPI literature [1, 2]. Many participatory frameworks implicitly assume stable availability of co-researchers over time [27, 28], while hospice, palliative and oncology care settings are often characterized by unpredictable disease trajectories, caregiving responsibilities, and emotional burden. Our findings therefore suggest that meaningful involvement should not be equated with maximal participation. Instead, flexible models of involvement may be better suited for populations affected by serious illness [1].

This need for flexibility was reflected in the adaptations implemented throughout both projects. Researchers modified participatory structures in response to barriers identified during the process, including limited engagement in written tasks, technical barriers, and varying availability of co-researchers. Practical strategies included flexible scheduling, facilitated working sessions, simplified digital tools, regular updates, and a stronger focus on verbal feedback and shared decision-making during workshops. These adaptations were not merely supportive measures but shaped concrete methodological decisions and research outputs. They demonstrate how PPI can influence the research process itself, an aspect that has often been insufficiently described in previous literature [29].

Another important finding concerns accessibility in participatory research. Co-researchers did not only encounter language-related barriers due to English-language scientific literature, but also struggled with scientific terminology, methodological concepts, and study designs. This points to a broader issue of epistemic accessibility. Similar knowledge asymmetries between researchers and public contributors have been described previously [18]. Knowles et al. [30] argue that this may create a risk whereby researchers instrumentalize co-researchers’ perceived lack of expertise to legitimize their own decisions, ultimately reinforcing tokenistic forms of involvement, described as “confirmation logic”. Our findings therefore highlight the importance of designing participatory processes that make scientific knowledge more accessible without reinforcing hierarchies between academic researchers and co-researchers.

Power imbalances were also evident within our heterogeneous groups of co-researchers. In multidisciplinary discussions, healthcare professionals, particularly physicians, sometimes dominated conversations, potentially limiting the contributions of patients and relatives. This finding illustrates that bringing different stakeholder groups together does not automatically create equitable participation [4, 18]. Differences in professional background, research experience, communicative confidence, and familiarity with scientific procedures shaped participation dynamics within the projects, potentially reinforcing existing power asymmetries [17]. While involving co-researchers with prior experience or formal expertise may facilitate project work, privileging these forms of knowledge risks reproducing existing hierarchies and marginalizing experiential perspectives [29, 31, 32]. However, these discussions primarily focus on knowledge hierarchies between researchers and co-researchers and raise the question of how differences in competencies and levels of professionalization should be addressed in projects that involve both patients and healthcare professionals (e.g., physicians) as co-researchers [33]. Our findings, however, underline the importance of actively fostering heterogeneity among co-researchers, even if this introduces additional complexity into the research process. In our view, the success of participatory research should not be measured solely in terms of individual contributions or perceived “accuracy” of statements. Instead, participatory processes should recognize different forms of knowledge as complementary and equally relevant for shaping research outcomes [27].

Based on our findings, we argue that active moderation by researchers is needed to ensure that diverse perspectives are heard and integrated into decision-making processes. At the same time, this highlights the need for tailored approaches in PPI that account for the heterogeneity of co-researchers and their respective needs [34]. Furthermore, researchers must be aware of the risks associated with participatory involvement and critically reflect on their own role within these collaborative settings. Defining and acknowledging their position within inherently non-linear and dynamic processes is essential to avoid reinforcing existing power imbalances [30, 35].

In summary, our findings underscore that meaningful PPI in oncology and palliative care research hinges on a dynamic balance between clear structural guidance and the flexibility to accommodate fluctuating capacities, diverse knowledge bases, and evolving relational dynamics [10]. In this sense, meaningful participation and involvement are not achieved by applying a fixed involvement model, but by continuously adapting participatory processes in ethically sensitive ways. For palliative care research in particular, this means designing PPI processes that enable contribution without overburdening co-researchers, support co-design without reinforcing hierarchies, and create conditions under which patient and public voices can genuinely shape research processes and outputs.

### Strengths and limitations

This study draws on experiences from two PPI projects conducted along the oncology care pathway (clinical and palliative), enabling the identification of both shared and context-specific challenges of PPI. The use of multiple qualitative data sources and thematic triangulation provided insights into participatory processes from both researcher and co-researcher perspectives. Furthermore, the study extends existing descriptive accounts of PPI by illustrating how co-researcher involvement influenced methodological decisions and project procedures.

Several limitations should be considered. Data sources differed between projects, with one project relying on reflective group discussions and the other primarily on process documentation and observational notes. In addition, both projects were conducted in Germany, which may limit transferability to settings with more established PPI infrastructures. Selection bias may have occurred, as co-researchers who remained involved throughout the projects may have differed from those who withdrew or were unable to participate. Finally, because researchers evaluated their own participatory processes, interpretative bias cannot be excluded, although triangulation was used to enhance the credibility of the findings.

## Conclusion

The findings of this evaluation of PPI across two research projects demonstrate that the involvement of patients and other stakeholders plays a crucial role in health services research, including within the context of oncological care pathways. However, the vulnerable life situations of individuals within these settings require particularly sensitive and flexible approaches to participation in order to ensure that their needs can be meaningfully and sustainably integrated into the research process. Our findings suggest that there is a need to reflect on how collaboration with patients, relatives, and healthcare professionals should be structured in palliative care research projects in order to minimize power imbalances. At the same time, co-researchers themselves can play an important role in shaping participatory processes by providing feedback that informs ongoing adaptations. These adaptations may at times involve unconventional approaches. However, we consider this flexibility to be a valuable contribution to the research process and encourage other researchers to more actively incorporate the perspectives of co-researchers when designing and conducting participatory research.

## Supplementary Information

Additional file 1.

## Data Availability

The datasets analyzed during the current study are not publicly available in order to protect the co-researchers involved in the projects but are available from the corresponding author on reasonable request.
