# Supplementary material for: Enabling meaningful participation and involvement in oncology and palliative care research: insights from two participatory research projects
Source: BMC Palliat Care. 2026 Nov 4;25:332. doi: 10.1186/s12904-026-02415-3 (PMC13737623; doi:10.1186/s12904-026-02415-3)
Supplement: Supplementary file 1 [file 12904_2026_2415_MOESM1_ESM.pdf]

Table 1. Overarching themes and subthemes identified in the retrospective qualitative process evaluation of participatory processes across SETT-AID and ParDiMi.

Overarching theme	Subthemes	Description
**Facilitating aspects**	• Motivation and perceived relevance<br>• Early involvement and influence<br>• Collaborative relationships<br>• Accessible and meaningful research tasks	Conditions that supported active participation, trust, ownership, and sustained engagement
**Challenging aspects**	• Changing participation capacities<br>• Scientific and epistemic complexity<br>• Digital and accessibility barriers; communication<br>• Power imbalances<br>• Adaptations of participation formats	Challenges emerging during collaboration and strategies used to respond to them
**Adaptive strategies**	• Flexible organization and adaptation of workload<br>• Tailored methodological and linguistic support<br>Accessible digital and technical<br>• Support<br>• Communication, feedback and facilitation of equitable participation	Strategies introduced or developed in response to emerging barriers and changing participation needs
**Perceived impacts of participation**	• Learning and confidence<br>• Agency and empowerment<br>• Relevance and usability of research processes and outputs	Perceived consequences of involvement for co-researchers and the research process
